# A Robust Neuromuscular Interface to Restore Lost Function in People with Amputations

**DOI:** 10.21203/rs.3.rs-5989030/v1

**Published:** 2025-05-14

**Authors:** Alex Vaskov, Dylan Wallace, Karan Desai, Ann Laidlaw, Theodore Kung, Deanna Gates, Stephen Kemp, Cynthia Chestek, Paul Cederna

**Affiliations:** University of Michigan-Ann Arbor; University of Michigan-Ann Arbor; University of Michigan-Ann Arbor; University of Michigan-Ann Arbor; University of Michigan-Ann Arbor; University of Michigan-Ann Arbor; University of Michigan-Ann Arbor; University of Michigan; University of Michigan-Ann Arbor

## Abstract

Upper limb loss can negatively impact an individual’s ability to perform daily tasks as well as mental health and well-being. Currently available prosthetic control interfaces provide limited prosthetic finger dexterity compared to the complex functions that multi-articulating robotic hands are capable of actuating. A significant barrier is the ability to reliably sense efferent motor action potentials from peripheral nerves when a patient’s muscles are lost or damaged due to amputation and injury. In an early-feasibility clinical trial, we implanted four patients with intramuscular electrodes in Regenerative Peripheral Nerve Interfaces (RPNIs). In all patients, the electrodes recorded large-amplitude and stable control signals from RPNIs with a median Signal-to-Noise Ratio (SNR) of 40.6 throughout their study participation. No serious adverse events occurred related to the electrode implantation or the devices themselves. Furthermore, implanting RPNIs provided valuable information to create an algorithm to predict movements previously mediated by lost muscles. These results indicate RPNI-electrode implantation is a repeatable and viable technique to record nerve signals for prosthetic control.

## Introduction

Upper limb amputations, often resulting from traumatic limb injuries, significantly impacts activities of daily living and can lead to depression and loss of employment ^1,2^. In 2017, it was estimated that 22.3 million people globally were living with major upper limb amputations due to traumatic causes ^3^. Despite advanced robotics, this patient population is not well-served due to limitations in accurate and reliable control of multiple degrees of freedom (DoFs) including individual fingers, wrist, and elbow control ^4,5^. Notably, as high as 44% of people with upper-limb amputations choose not to use a prosthetic device, but among those who do, most prefer a myoelectric prosthesis ^6–10^. The current standard of care for myoelectric prostheses is dual-site control in which an agonist-antagonist muscle pair controls a single DoF ^11^. However, this paradigm does not translate well from the muscle pair’s physiological function to prosthetic function, for example using elbow movements to control the wrist is neither intuitive nor naturalistic. To control multiple functions with only two control signals, users must cycle through movements and pre-programmed grips using muscle activation sequences or specific programmed body movements, such as with the arm or foot, to trigger transitions ^12,13^. The aforementioned control interfaces remain cumbersome and unintuitive ^14,15^.

Increasing the number of EMG channels can capture increased information from the residual limb, enabling more seamless activation of multiple functions ^16–22^. Pattern recognition systems typically use eight channels of surface EMG and machine learning algorithms to categorize the signals into elbow, wrist, and hand movements. This technology has benefited some users and outperformed dual-site control in various functional tests ^20,23^. Reliability remains a concern as recalibration is often required when wearing or removing the prosthesis, and shifting arm position can diminish controller accuracy ^24–27^. Commercial systems are also limited to the sequential activation of single movements and pre-programmed grips. While increased the number of electrodes can enable more precise and dexterous movements with surface EMG, many studies have used able-bodied participants, raising concerns about reliability for multi-DoF control and generalizability to people with major limb amputations ^28–30^. It is only possible to record EMG from innervated muscles which remain in the residual limb. If there are no remaining residual innervated muscles in the limb to control the wrist or fingers (i.e. above elbow amputation), there are very few control signals to provide multi-DOF control. For this reason, there is a limit to the amount of control that can be achieved with surface EMG strategies. Surgically implanting intramuscular or epimysial electrodes in residual innervated muscles has been shown to capture strong and reliable EMG signals ^31–34^. Implanted EMG electrodes improve control accuracy and reduce movement variability compared to surface EMG ^31,34–37^. Lukyanenko et al. demonstrated control of 4 DoF without the need for controller recalibration ^37^. However, the patient in that study used a substituted movement (wrist deviation) to control thumb opposition. This highlights the need to interface with peripheral nerves as well as muscles to restore naturalistic thumb function.

To address limitations of surface and intramuscular EMG, multi-contact penetrating electrodes that directly record efferent nerve action potentials have been explored ^38–42^. This has enabled proportional and simultaneous control of multiple DoFs of individuated fingers with high specificity ^32,41–44^. However, achieving long-term stability with nerve recordings has proved challenging with the most prolonged stable peripheral nerve interface lasting 16 months in a person with limb loss, and only 9% of electrodes remaining functional at that point ^45^. Other direct nerve recording methods face similar hurdles, including issues with nerve specificity, tissue injury, axonal degeneration, and scar tissue attributed to chronic foreign body response ^46^. These issues limit the long-term viability of these implants for motor control. Only a few studies have shown that electrodes implanted into muscles reinnervated by targeted muscle reinnervation (TMR) can provide missing signals for prosthetic control ^33,47^. However, in clinical practice, TMR has been limited by signal resolution and reduced reliability due to use of surface EMG ^48^. This is not a function of the TMR procedure but rather a function of the use of surface strategies to control prosthetic movements. In addition, the signal resolution for TMR could be improved if the TMR procedure is refined to provide more individual, discrete motor control signals.

Our group developed the Regenerative Peripheral Nerve Interface (RPNI) to enhance prosthetic function by creating additional control sites and capturing lost efferent motor activity from severed peripheral nerves ^49–53^. The RPNI involves surgically implanting the distal end of a transected nerve into an autogenous free muscle graft, promoting axonal sprouting, elongation, and reinnervation within 8 weeks ^49,52,54^. Efferent motor action potentials stimulate RPNI contraction, amplifying efferent motor activity into large EMG signals ^49,53,54^. Previous studies showed that implanting intramuscular electrodes into RPNIs captures high signal-to-noise ratio (SNR) EMG up to 5 years post-RPNI creation. Combining RPNI signals with implanted residual innervated muscles has enabled patients to control multiple DoF, including precise thumb movements, and reliably control multiple hand functions without the need for persistent recalibration ^50,53,55,56^.

Here we report the interim results of our clinical trial. The aim of this early feasibility study was to assess the safety and efficacy of implanting electrodes into RPNIs for enhanced prosthetic control. Four patients with transradial amputations underwent surgery to implant bipolar intramuscular electrodes in 14 RPNIs and 26 residual innervated muscles. Monthly SNR measurements showed RPNIs produced large amplitude EMG with a median SNR of 40.6 (32.2 dB). Additionally, RPNIs demonstrated stability over time, with no decreasing trend in SNR and some even increasing in strength. Incorporation of the RPNI signals as a control input reduced classification error of independent thumb movements and finger ab/adduction by 15.5% on average, over use of control signals from residual innervated muscles alone. Implanting electrodes into RPNIs is also shown to be a safe procedure, with only four minor adverse events occurring in over 115 patient-months. There were no increases in levels of neuropathic or phantom limb pain. These findings indicate that implanting electrodes into RPNIs is a viable long-term solution to restore function following limb loss.

## Results

### RPNIs Are a Biologically Stable Nerve Interface

**Figure 1:**
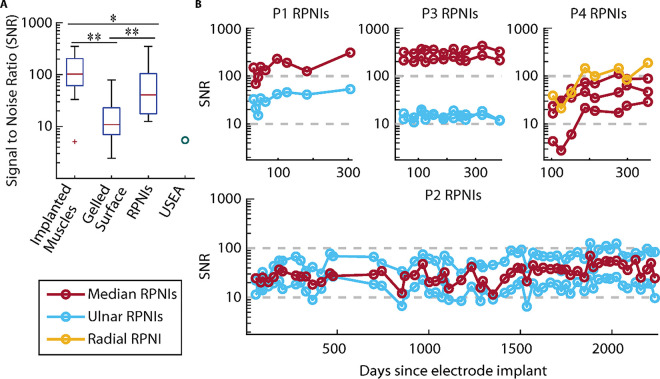
RPNI signal strength and stability. (**A**) Signal to Noise Ratio (SNR) of RPNI activation during preferred movements over time. (**B**) Comparison of SNRs between implanted residual muscles (n=26, P1-P4), gelled surface electrodes (n=16 channels, P2 and P4), RPNIs (n=13, P1-P4), and average SNR of a Utah Slant Electrode Array (USEA, ^45^).

Four patients with transradial amputations had bipolar intramuscular electrodes surgically implanted into RPNIs and residual innervated muscles for prosthetic control. Bipolar EMG signals were recorded from 13/14 RPNIs and 26/26 residual muscles across the four patients. Evidence indicates the 14^th^ RPNI successfully reinnervated, however, there was a connection issue with the negative implanted electrode contact (see *Supplementary Materials*, Fig. S1). During the study, signal-to-noise ratios (SNRs) were calculated monthly by comparing the EMG signal strength during volitional finger movements of their phantom limb, to baseline noise. Figure 1a shows that the implanted RPNIs produced large amplitude EMG with a median SNR of 40.6 (32.2dB, n = 13), representing an average signal strength 7.5 times that of nerve electrodes found in literature. The same intramuscular electrodes implanted in RPNIs were also implanted in residual innervated muscles and recorded EMG with a median SNR of 102.3 (40.2dB, n = 26). Two patients (P2 and P4) each had eight gelled, surface EMG electrodes placed simultaneously with the implanted electrodes to compare recorded signals amplitude. Implanted electrode signals from both RPNIs and residual innervated muscles were significantly stronger (p < 0.01, Wilcoxon rank-sum test) than the highest amplitude surface EMG signals, which had a median SNR of 10.9 (20.7dB, n = 16 channels).

As shown in Figure 1b, RPNI signaling remained stable over time. Three patients (P1-P3) had RPNIs surgery performed 1.0 – 4.3 years before electrodes were implanted in their median and ulnar nerve RPNIs. P4 had electrodes implanted in his radial and median nerve RPNIs at the time of RPNI creation. In all of these patients, no RPNIs showed a decreasing trend in SNR over time (p > 0.05, F-test). SNRs for P1, P3, and P4 were measured up to 306, 384, and 354 days after electrode implantation, respectively. P2 had SNRs measured 2,234 days post-implant at the time of this report. P1’s RPNIs had a significant SNR increase over time (p < 0.05, F-test), indicating RPNIs have the potential to continue to increase in EMG amplitude over time. All of P4’s RPNIs also displayed a significant and more dramatic increasing trend over time, which is expected because unlike P1-P3, his RPNIs were created simultaneously with electrode implantation. We began SNR measurements 103 days after his surgery based on the 3-month reinnervation period observed in rodents. In his first SNR session, EMG was detectable (average SNR of 20.1), but substantially increased by 188 days (average SNR of 60.3). These results indicate that implanting electrodes into RPNIs is a biologically stable method to record amplified efferent action potentials from peripheral nerves.

### RPNIs Restore Lost Function

**Figure 2:**
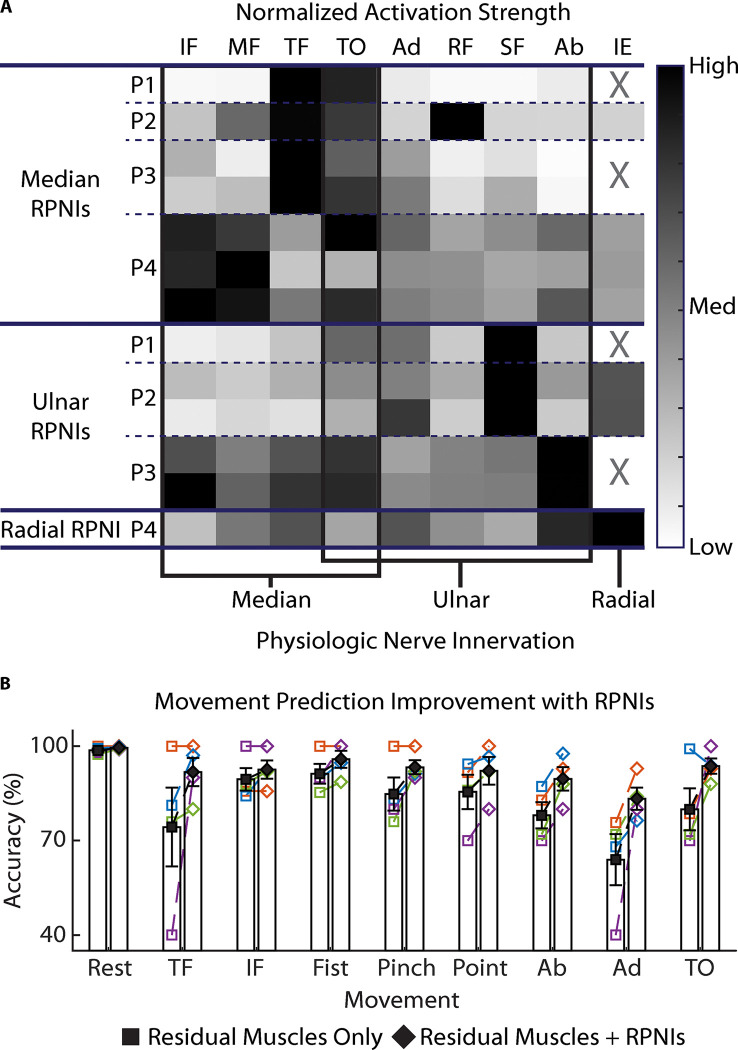
RPNI preferred movements. (**A**) Normalized activation strength across Thumb, Index, Middle, Ring and Small Finger Flexion (TF, IF, MF, RF, SF), Thumb Opposition (TO), Finger Ab/Adduction (Ab/Ad), and Index Finger Extension (IE). “X” indicates no data collected. Movements are ordered by physiologic innervation. (**B**) Including RPNIs as a control input improves distinction of individual finger and thumb movements from Rest (no motion) and multiple grasps (Fist, Pinch, Point). Filled shapes and bars (mean±s.e.m.) summarize individual data (P1 – orange, P2 – blue, P3 – purple, and P4 – green) for each movement.

In all four patients, the RPNI signals produced in response to phantom movements were generally expected based on prior anatomy. For example, as shown in Figure 2a, P1’s Median RPNI selectively activated for thumb flexion and thumb opposition. Across all four patients, the majority of both median and ulnar RPNIs activated during thumb opposition, consistent with the median and ulnar nerves’ physiologic innervation of muscles within the hand that control thumb opposition and rotation. Additional intrinsic hand muscles control finger ab/adduction and metacarpophalangeal joint flexion. It is not surprising that RPNIs activate during multiple hand functions. In some cases, the response to movements was not physiologically expected. For example, one of P3’s ulnar RPNIs activated strongly during index flexion. This could be a behavior in which a missing intrinsic hand muscle is co-activated during index flexion. In most cases, when a nerve was divided to create multiple RPNIs, the strongest movements were the same (see Materials and Methods). However, the individual RPNIs had different activation profiles across finger movements. These differing patterns can be useful for prosthetic control algorithms to distinguish independent movements.

In transradial amputations many of the muscles used to control the wrist and fingers are still present and can be implanted with electrodes to facilitate prosthetic control. The extrinsic muscles of the hand are anatomically located in the forearm and can be used for prosthetic control by implanting electrodes into them. The intrinsic muscles of the hand are absent since they are anatomically located in the hand. As a result, to control prosthetic function in these cases, the electrodes will need to be implanted into RPNIs, since no residual muscles are present. To evaluate the benefit of implanting RPNIs at this amputation level, we performed a post-hoc analysis to identify which movements RPNIs were most valuable at predicting. For each patient, a movement classifier was trained to predict nine movements: 1) rest (no motion); 2) three hand grasp patterns controlled by extrinsic muscles of the hand (electrodes implanted into the flexor digitorum profundus muscle to the index finger and the flexor policis longus muscle to the thumb); and 3) movements controlled by intrinsic hand muscles (electrodes implanted in median and ulnar nerve RPNIs to control finger abduction, finger adduction, and thumb opposition). The impact of RPNI signals was determined by comparing classifier error rates with and without RPNIs as a control input (Fig. 2b). The most positively impacted movements were thumb flexion, thumb opposition, and finger adduction. When RPNIs were added as a control input, their respective prediction accuracies increased to 92.1% (17.5% improvement), 93.6% (13.7% improvement), and 83.8% (19.4% improvement). Meanwhile RPNIs only improved distinction of index finger flexion by 3.1% and grasp patterns by 6.6% on average. These results indicate that RPNIs contributed most to distinguishing movements controlled by absent intrinsic thenar, hypothenar, and interosseus muscles.

### Safety of Electrode Implantation

The bipolar electrodes used in this study were percutaneous electrodes, exiting the skin either through the residual forearm (P1) or upper arm (P2-P4). The patient was instructed to follow a weekly exit site cleaning protocol. During in-lab experiments, the exit site cleaning was performed by study team members. In between uses, the exit sites were covered with a gauze pad secured by an occlusive Tegaderm film dressing (3M). As shown in Table 1, only four non-serious adverse events have occurred related to the electrode implant. In two cases, the patient had a very mild skin infection which was treated by a short course of oral antibiotics and resolved. The lack of serious infections with a long-term percutaneous interface is consistent with literature that reports low complication rates using similarly designed percutaneous leads in other applications. We suspect this is due to the small lead diameter (0.75mm) and exposed coil design, which creates small caliber exit sites and minimizes pistoning. No instances of electrode extrusion or migration were observed. In two other non-serious adverse events, one patient experienced a reaction to the Tegaderm dressing and one patient had bruising from donning and doffing he socket. Both of these cases resolved spontaneously without any medical intervention. Four non-serious and two serious adverse events have occurred unrelated to the study.

**Figure 3:**
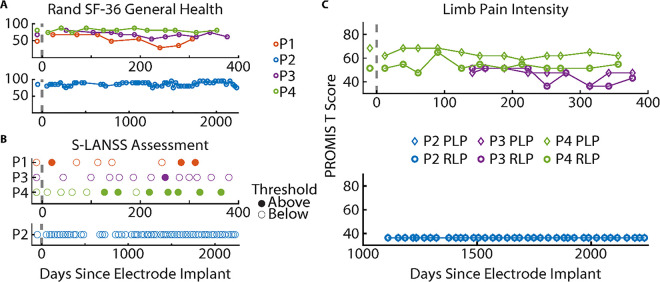
Self-reported quality of life and pain assessments. (**A**) Rand 36-Item Short Form (SF 36) survey scores for general health. (**B**) Self-administered Leeds Assessment of Neuropathic Symptoms and Signs (S-LANSS). (**C**) A limb pain survey used the Patient-Reported Outcomes Measurement Information System (PROMIS) Short Form 3A to measure the intensity of phantom limb pain (PLP) and residual limb pain (RLP). This survey was added midway through the study after P1’s withdrawal.

All four patients filled out monthly survey instruments to assess changes in general health and pain levels (Fig. 3). Results of the Rand 36-Item Short Form did not indicate decreasing trends in general health (p > 0.05, F-test). The Self-administered Leeds Assessment of Neuropathic Symptoms and Signs (S-LANSS) survey was given to detect neuropathic pain throughout the study. P1-P3 showed little-to-no instances of neuropathic pain resulting from implantation. P4 reported the unchanged presence of phantom limb pain prior to and during his time in the study. His S-LANSS score was below the neuropathic pain threshold during pre-screening, yet he indicated the presence of neuropathic pain 7/12 times throughout the study. This is not surprising as phantom limb pain likely has a neuropathic component. A limb pain survey was added to the protocol prior to his enrollment. This survey asked patients to separately rate the intensity of both phantom and residual limb pain using the Patient-Reported Outcomes Measurement Information System (PROMIS) Short Form 3a. These results indicated no increasing or decreasing trend in the intensity of P4’s limb pain (p > 0.05, F-test), which was categorized as moderate based on the PROMIS T-Scores.

## Discussion

Intermediate results of this clinical trial show that implanting electrodes into RPNIs is a safe and effective way to interface with peripheral nerves for prosthetic control. Four patients received 14 RPNI-electrode implants which appeared to remain healthy, evidenced by stable efferent signaling, and there were no observed changes in neuroma or phantom limb pain. The four patients had an age range of 33–74 and were implanted with electrodes 1.0–4.7 years after amputation. P1, P3, and P4 remained implanted in the study for 11.9–17.0 months, while P2 has been in the study for over 6 years at the time of this report. The loss of thumb function results in a 40–50% decrease in hand function ^57,58^. Here we show that at a transradial level, implanting electrodes into RPNIs, in addition to residual muscles, provides valuable signals to restore independent thumb movements. The thumb function is controlled by both extrinsic muscles of the hand like the flexor policis longus (FPL) and the extensor policis longus (EPL) and intrinsic muscles of the hand including the flexor policis brevis (FPB), opponens policis (OP), and adductor policis (AP). To have optimal thumb control, input from the extrinsic muscles (electrodes implanted into the residual innervated muscles) and intrinsic muscles of the hand (electrodes implanted into RPNI), can be combined and integrated to provide high fidelity control. In more proximal amputations, acute experiments show that implanting RPNIs can provide valuable control signals for finger flexion and extension in the absence of extrinsic hand muscles ^50^. Importantly, the results achieved here are consistent with other studies that implanted EMG electrodes into innervated and reinnervated muscle tissue. Zbinden et al. implanted intramuscular electrodes into RPNIs and demonstrated stable signaling for prosthetic control over an observed period of 2.4 years ^47^. That study and others have shown that intramuscular electrodes can also be implanted in TMR constructs to record amplified signals from peripheral nerves. Optimizing implantable interfaces for TMR and RPNI cases is important as both surgeries are commonly performed to prevent or treat limb pain. To further increase signal resolution, P2-P4 had their ulnar and/or median nerves divided to create multiple RPNIs on a single nerve. Future studies and clinical experience are needed to develop guidelines on the optimal number of RPNIs to create and implant at different amputation levels. Collectively, this research supports implanting EMG electrodes into reinnervated muscle tissue as a promising method to biologically amplify nerve signals and restore function after limb loss.

Prior research confirms that both TMR and RPNI surgery are highly effective in treating residual limb pain resulting from neuroma formation ^59–63^. Overall, the results here indicate that implanting electrodes into RPNIs does not diminish this benefit. In three patients (P1-P3), electrodes were implanted in a secondary surgery 1.0–4.3 years after RPNIs were created for the purpose of treatment or preventing neuroma and phantom limb pain. In these cases, the RPNI graft had ample time to reinnervate and mature before electrode implantation. P4 had RPNIs created and electrodes implanted in the same operation. The observed increase in SNR indicates that P4’s RPNIs reinnervated and remained healthy, demonstrating that RPNI surgery and electrode implantation may be consolidated into a single operation. This may reduce the number of operations a patient undergoes and correspondingly reduce the potential complications. Although P4 verbally reported decreased pain after the study intervention, his self-administered surveys indicated the presence of neuropathic pain and no significant change in the intensity of residual and phantom limb pain throughout the study. P4 had a previous TMR operation that failed to resolve his neuroma pain and his phantom limb pain prior to his enrollment in this study. Based on the quantitative data from one unique case, we cannot definitively state whether or not consolidating RPNI surgery and electrode implantation into a single operation impacts the safety profile of the procedure. Additionally, while RPNI and TMR are highly effective at treating localized residual limb pain, phantom limb pain persists in some cases. The true underlying mechanisms of phantom pain are not well understood, however many theories suggest a disruption of central and peripheral sensorimotor circuitry ^64,65^. Recent studies have investigated the effects of exercises that promote volitional hand control via phantom motor execution as well as transcutaneous electrical stimulation to alleviate phantom limb pain ^66,67^. Implanted devices can perform similar functions during regular prosthesis use and anecdotal evidence and case studies suggest this may play a role in reducing limb pain ^68–70^. Future studies should evaluate limb pain with daily use of advanced control and feedback interfaces to determine if there is a clinically significant benefit.

The primary goal of this early feasibility study was to determine the safety and efficacy of implanting electrodes into RPNIs for enhanced prosthetic control. Gonzalez et al. recently reported on a secondary endpoint of the study, demonstrating stable afferent signaling of RPNIs ^71^. In all four patients, electrically stimulating RPNIs produced somatotopically accurate cutaneous sensory feedback with distinguishable intensities. Monthly measurements of the charge threshold required to perceive a sensation demonstrated afferent signaling were mostly either stable from initial measurements or stabilized within 6 months. Sensory feedback is a critical aspect of control in any engineering system. Many users of myoelectric prostheses are frustrated by the lack of sensory feedback ^9^. Although implantable EMG electrodes can greatly improve feedforward control, achievable dexterity may be limited by a lack of feedback. Providing kinesthetic feedback of joint movement has been shown to improve motor performance ^72^. Increasing the amplitude of RPNI stimulation can produce finger movement sensations in addition to cutaneous feedback ^71,73^. Other studies have also shown that providing tactile sensory feedback via electrical stimulation improves fine motor control and prosthesis embodiment ^70,74–76^. To date, the results of this clinical trial have shown that RPNIs and implanted electrodes are a safe and promising technique to improve functional restoration for people with upper limb loss. We anticipate that take-home trials of ours and similar technologies will reveal multiple factors that improve healthcare outcomes for people with upper limb amputations.

## Materials And Methods

### Study Design

Four patients (P1, P2, P3, and P4) were enrolled in the early-feasibility study (clinicaltrials.gov NCT03260400) and proceeded with the electrode implantation procedure. A fifth patient was enrolled in the study, but was withdrawn prior to electrode implantation after subsequent screening and evaluation of comorbidities. All patients provided informed consent for the study procedures which were approved by the University of Michigan Institutional Research Board (HUM00124839).

### Participant Demographics and Anatomy

Patients were implanted with either 8 (P1 and P2) or 12 (P3 and P4) percutaneous electrodes. The electrodes were a modified version of the long-term electrodes used in the NeuRx^®^ diaphragmatic pacing system (Synapse Biomedical, PMA P200018). Patient demographics and implant surgery are summarized in Table 2. While the inclusion/exclusion criteria specified candidates shall be ASA Class I or II, the causes of amputation varied between patients. P1 sustained a traumatic amputation of the right hand, resulting in a wrist disarticulation. The patient subsequently underwent a transradial amputation with RPNIs to treat their neuroma pain and phantom limb pain. P2 had a partial right hand amputation as a result of necrotizing fasciitis. The patient experienced significant neuroma pain and phantom limb pain along with limited range of motion. P2 voluntarily underwent a distal transradial amputation with RPNI. P3 underwent a transradial amputation with prophylactic RPNIs to treat a sarcoma. The patient was not enrolled in the study until they were deemed cancer free after 2 years. P4 sustained a traumatic amputation of the hand resulting in a wrist disarticulation. Prior to his enrollment, he had Targeted Muscle Reinnervation surgery and it was later discovered that his ulnar nerve was transected above the elbow. He did not experience any relief in his pain following his TMR. At the time of his enrollment in the study, the patient elected to have the limb shortened during his study operation to incorporate an electronic wrist rotator in his personal prosthesis. RPNIs were also performed at the same time as electrode implantation.

P1, P3, and P4 were all withdrawn from the study for various reasons. P1 had a change of employment and did not wish to maintain the percutaneous electrode exit sites at his new job. P3 had a cancer recurrence in his hip and withdrew from the study. P4 unexpectedly passed away from a heart-attack. These unfortunate events were unrelated to the study intervention. P2 remains implanted and actively participating in the study.

### Signal Strength Measurements

The percutaneous electrodes were connected to a neural signal processor (NeuroPort, Blackrock Microsystems) and Matlab target xPC for analysis. The neural signal processor recorded bipolar EMG at 30 kSps. A Matlab target xPC (Mathworks) bandpass filtered the EMG from 100 to 500 Hz and down-sampled the recording to 1 kSps. Signal-to-noise ratios were collected on a monthly basis, with some deviations due to patient availability and a temporary pause in experiments due to COVID 19. The xPC computer time-synced EMG recordings to a laptop with a virtual display that prompted patients to make movements with their phantom hand. For each channel, SNR was calculated by dividing the root mean square (RMS) of the filtered EMG during volitional phantom movements by the RMS of the electrode’s noise floor during no movement. In all cases, RPNIs activated for multiple hand and finger movements. The movement cue that generated the strongest response SNR for RPNI (Table 3) was used to analyze signal stability. P1 completed 9 SNR sessions across 276 days (30 to 306 days post-implant), P2 has completed 58 sessions across 2179 days (55 to 2234 days post-implant), P3 completed 10 sessions across 335 days (49 to 384 days post-implant), and P4 completed 8 sessions across 251 days (103 to 354 days post-implant). The strongest hand and wrist movement for each channel was also used to compare signal strengths between implanted residual muscles and P2 and P4’s surface EMG (Biopac Ag/AgCl adhesive electrodes with conductive gel). SNRs were used as the primary indicator of RPNI tissue health due to imaging difficulties described in *Supplementary Materials*.

### RPNI Preferred Finger Movements and Movement Prediction Analysis

To visualize the activation profiles of each RPNI, the average activation RMS was normalized across individual finger movements for each RPNI. Data for the following movements was collected for all patients: thumb flexion, index finger flexion, middle finger flexion, small finger flexion, thumb opposition, finger abduction and finger adduction. Individual index finger extension data was only collected for P4 and the later part of P2’s study participation. The ground truth of movement cannot be measured in the case of amputation and some cues may generate coupled movement responses. This is likely due to naturally occurring muscle synergies in some cases. For example, it is difficult to fully abduct the fingers without also extending the fingers and thumb. The response to finger abduction likely represents these combined movements. Coupled movements may also differ between patients due to differences in phantom limb awareness, coordination, and behavior.

To determine the benefit of implanting RPNIs at a transradial level, a post-hoc analysis compared the prediction accuracy of two Linear Discriminant Analysis classifiers that were trained to predict nine movements: rest (no movement), thumb flexion, index flexion, fist, pinch, point, finger abduction, finger adduction, and thumb opposition. These movements represent individual and combined finger and thumb movements that are controlled by both intrinsic and extrinsic muscles of the hand, which all patients had implanted. The first classifier was trained with signals from only residual hand and wrist muscles. RPNIs were added as an input to the second classifier. The classifiers were individually calibrated for each patient and session. Average trial performance was simulated using leave-one-out cross validation. The analysis was run on 2 sessions for P1, 24 sessions for P2, 1 session for P3, and 5 sessions for P4, varying based on data availability. RPNIs were inferred to be valuable to predict movements which saw the greatest improvement in performance.

### Survey Administration

Throughout the study, pain levels and quality of life were tracked using self-administered surveys that were collected on a monthly frequency, with some deviations due to patient availability. General health and quality of life were tracked using the Research and Development 36 item short form survey (RAND SF-36). The self-assessed Leeds Assessment of Neuropathic Symptoms and Signs (S-LANSS) was used to determine the presence of neuropathic pain. Later in the study, a Phantom Limb Pain survey was added to measure intensity of both residual and phantom limb pain. The Phantom Limb Pain survey used the Patient Reported Outcomes Measurement Information System for pain intensity (PROMIS pain intensity 3a) to independently measure the intensity of phantom and residual limb pain. Surveys were administered during lab visits and patients were instructed to answer the questions to the best of their ability. Scoring for each of the surveys was conducted with their respective standards.

### Statistical Analysis

Differences between the Signal to Noise Ratio (SNR) of implanted residual muscles (n = 26), RPNIs (n = 13), and surface EMG (n = 16) were assessed using a Wilcoxon rank-sum test. Trends in the SNR of RPNIs over time were analyzed with linear regression model and F-test for a non-zero slope. Trends in general health (RAND SF-36 score) and limb pain intensity (PROMIS 3A T-score) over time were also analyzed with linear regression model and F-test for a non-zero slope. Neuropathic pain (S-LANSS) was not statistically analyzed since it is a threshold-based determination and there were no occurrences in any patient’s pre-op session. Movement prediction accuracy with and without RPNIs was not statistically compared due to the low sample size (n = 4 patients).

## Figures and Tables

**Figure 4:**
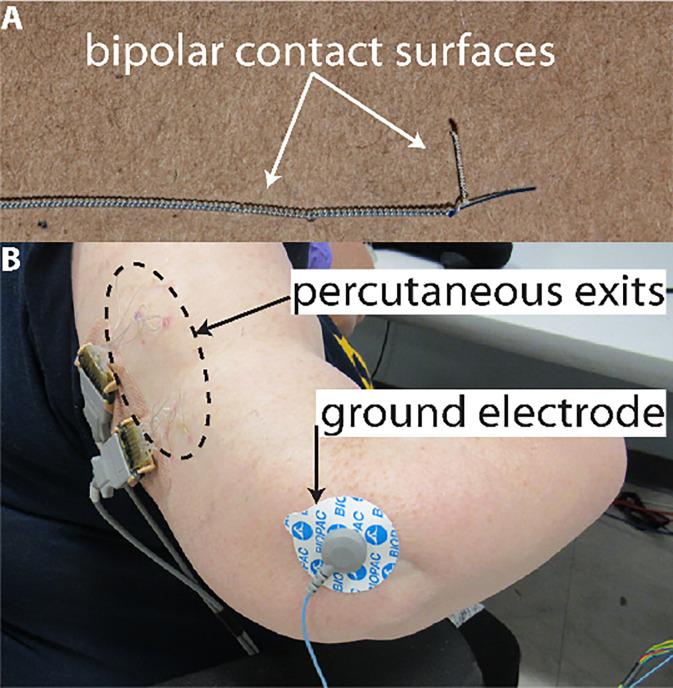
Surgically implanted intramuscular electrodes used in this study. (**A**) bipolar contact surfaces recorded differential signals. (**B**) percutaneous electrode exits and external ground (P2 shown).

**Table 1.** Adverse events related to electrode implant (^+^still in study). Two minor adverse events occurred related to other study procedures, and four unrelated adverse events have occurred.

Participant	Age	Number of Electrodes	Implant Duration (months)	Electrode-Related Adverse Events	
				Minor	Serious
P1	33	8	12.9	0	0
P2	52	8	73.4^+^	2	0
P3	74	12	17.0	1	0
P4	52	12	11.9	1	0

**Table 2:** Participant demographics at the time of electrode implant surgery. P2 and P3 had their RPNIs created at the time of amputation. P4 had electrodes implanted into newly created RPNIs in the same operation. In addition to RPNIs, a variety of residual hand and wrist muscles were also implanted for prosthetic control: Flexor Pollicis Longus (FPL), Flexor Digitorum Profundus to Index Finger (FDP-I), Flexor Digitorum Profundus to Small Finger (FDP-S), Extensor Pollicis Longus (EPL), Extensor Digitorum Communis (EDC), Flexor Carpi Radialis (FCR), Pronator Teres (Pronator), Extensor Carpi Radialis Longus (ECRL), and Supinator.

Patient Demographics			Electrode Implant Information			
ID	Gender	Age	Time since amputation (years)	Time since RPNI surgery (years)	Implanted RPNIs	Implanted residual muscles
P1	M	33	4.7	4.3	1 Median, 1 Ulnar	FPL, FDP-I, FDP-S, EPL, EDC, FCR
P2	F	52	1.0		1 Median, 2 Ulnar	FPL, FDP-I, EPL, EDC, FCR
P3	M	74	2.5		2 Median, 2 Ulnar	FPL, FDP-I, EPL, EDC, FCR, Pronator, ECRL, Supinator
P4	M	52	2.3	N/A	4 Median, 1 Radial	FPL, FDP-I, FDP-S, EPL, EDC, Pronator, Supinator

**Table 3:** Phantom movements use to track RPNI signal strength over time. Bipolar EMG could not be recorded for one RPNI (N/A), see Supplementary Materials for more information.

Patient ID	RPNI	Strongest Movement
P1	Median	Thumb Flexion
	Ulnar	Small Finger Flexion
P2	Median	Thumb Flexion
	Ulnar 1	Small Finger Flexion
	Ulnar 2	Small Finger Flexion
P3	Median 1	Thumb Flexion
	Median 2	Thumb Flexion
	Ulnar 1	Wrist Flexion
	Ulnar 2	Wrist Flexion
P4	Median 1	Thumb Opposition
	Median 2	N/A (hardware issue)
	Median 3	Wrist Flexion
	Median 4	Wrist Pronation
	Radial	Index Finger Extension

## Data Availability

Data used in this study is available upon reasonable request. Please contact Cynthia Chestek for data requests at cchestek@umich.edu.

## References

[1] Darnall B. D. Depressive symptoms and mental health service utilization among persons with limb loss: Results of a national survey. Archives of Physical Medicine and Rehabilitation 86, 650–658 (2005). 15827913 10.1016/j.apmr.2004.10.028

[2] Jang C. H. A survey on activities of daily living and occupations of upper extremity amputees. Annals of rehabilitation medicine 35, 907–921 (2011). 22506221 10.5535/arm.2011.35.6.907 PMC3309384

[3] McDonald C. L., Westcott-McCoy S., Weaver M. R., Haagsma J. & Kartin D. Global prevalence of traumatic non-fatal limb amputation. Prosthetics and orthotics international 45, 105–114 (2021). 33274665 10.1177/0309364620972258

[4] Ison M. & Artemiadis P. The role of muscle synergies in myoelectric control: trends and challenges for simultaneous multifunction control. Journal of neural engineering 11, 51001 (2014). 10.1088/1741-2560/11/5/051001 25188509

[5] Young A. J., Smith L. H., Rouse E. J. & Hargrove L. J. A comparison of the real-time controllability of pattern recognition to conventional myoelectric control for discrete and simultaneous movements. Journal of NeuroEngineering and Rehabilitation 11, 5 (2014). 24410948 10.1186/1743-0003-11-5 PMC3895741

[6] Pinzur M. S., Angelats J., Light T. R., Izuierdo R. & Pluth T. Functional outcome following traumatic upper limb amputation and prosthetic limb fitting. The Journal of hand surgery 19, 836–839 (1994). 7806814 10.1016/0363-5023(94)90197-X

[7] McFarland L. V., Hubbard Winkler S. L., Heinemann A. W., Jones M. & Esquenazi A. Unilateral upper-limb loss: satisfaction and prosthetic-device use in veterans and servicemembers from Vietnam and OIF/OEF conflicts. Journal of rehabilitation research and development 47, 299–316 (2010). 20803400 10.1682/jrrd.2009.03.0027

[8] Berke G. M. Comparison of satisfaction with current prosthetic care in veterans and servicemembers from Vietnam and OIF/OEF conflicts with major traumatic limb loss. Journal of rehabilitation research and development 47, 361–371 (2010). 20803404 10.1682/jrrd.2009.12.0193

[9] Biddiss E. & Chau T. Upper-limb prosthetics: Critical factors in device abandonment. American Journal of Physical Medicine and Rehabilitation (2007) doi:10.1097/PHM.0b013e3181587f6c. 18090439

[10] Raichle K. A. Prosthesis use in persons with lower- and upper-limb amputation. Journal of rehabilitation research and development 45, 961–972 (2008). 19165686 10.1682/jrrd.2007.09.0151 PMC2743731

[11] Roche A. D., Rehbaum H., Farina D. & Aszmann O. C. Prosthetic myoelectric control strategies: a clinical perspective. Current Surgery Reports 2, 1–11 (2014).

[12] Resnik L., Klinger S. L. & Etter K. The DEKA Arm: Its features, functionality, and evolution during the veterans affairs study to optimize the DEKA Arm. Prosthetics and Orthotics International (2014) doi:10.1177/0309364613506913. 24150930

[13] Franzke A. W. Users’ and therapists’ perceptions of myoelectric multi-function upper limb prostheses with conventional and pattern recognition control. PLoS ONE (2019) doi:10.1371/journal.pone.0220899. PMC6715185 31465469

[14] Heerschop A., van der Sluis C. K., Otten E. & Bongers R. M. Looking beyond proportional control: The relevance of mode switching in learning to operate multi-articulating myoelectric upper-limb prostheses. Biomedical Signal Processing and Control 55, 101647 (2020).

[15] Vilarino M. Outcomes and Perception of a Conventional and Alternative Myoelectric Control Strategy: A Study of Experienced and New Multiarticulating Hand Users. JPO: Journal of Prosthetics and Orthotics 27, (2015). 10.1097/jpo.0000000000000055 PMC10947723 38500562

[16] Hudgins B., Parker P. & Scott R. N. A New Strategy for Multifunction Myoelectric Control. IEEE Transactions on Biomedical Engineering (1993) doi:10.1109/10.204774. 8468080

[17] Ajiboye A. B. & Weir R. F. A heuristic fuzzy logic approach to EMG pattern recognition for multifunctional prosthesis control. IEEE Transactions on Neural Systems and Rehabilitation Engineering 13, 280–291 (2005). 16200752 10.1109/TNSRE.2005.847357

[18] Kuiken T. A. Targeted muscle reinnervation for real-time myoelectric control of multifunction artificial arms. JAMA - Journal of the American Medical Association (2009) doi:10.1001/jama.2009.116. PMC3036162 19211469

[19] Birdwell J. A., Hargrove L. J., Weir R. F. ff & Kuiken T. A. Extrinsic finger and thumb muscles command a virtual hand to allow individual finger and grasp control. IEEE transactions on biomedical engineering 62, 218–226 (2015). 25099395 10.1109/TBME.2014.2344854 PMC4501427

[20] Hargrove L. J., Miller L. A., Turner K. & Kuiken T. A. Myoelectric Pattern Recognition Outperforms Direct Control for Transhumeral Amputees with Targeted Muscle Reinnervation: A Randomized Clinical Trial. Scientific reports 7, 13840 (2017). 29062019 10.1038/s41598-017-14386-w PMC5653840

[21] Hahne J. M., Markovic M. & Farina D. User adaptation in Myoelectric Man-Machine Interfaces. Scientific Reports 7, 4437 (2017). 28667260 10.1038/s41598-017-04255-x PMC5493618

[22] Robertson J. W., Englehart K. B. & Scheme E. J. Effects of Confidence-Based Rejection on Usability and Error in Pattern Recognition-Based Myoelectric Control. IEEE Journal of Biomedical and Health Informatics 23, 2002–2008 (2019). 30387754 10.1109/JBHI.2018.2878907

[23] Resnik L. Evaluation of EMG pattern recognition for upper limb prosthesis control: a case study in comparison with direct myoelectric control. Journal of NeuroEngineering and Rehabilitation 15, 23 (2018). 29544501 10.1186/s12984-018-0361-3 PMC5856206

[24] Geng Y., Zhang F., Yang L., Zhang Y. & Li G. Reduction of the effect of arm position variation on real-time performance of motion classification. Annual International Conference of the IEEE Engineering in Medicine and Biology Society. IEEE Engineering in Medicine and Biology Society. Annual International Conference 2012, 2772–2775 (2012). 10.1109/EMBC.2012.6346539 23366500

[25] Hwang H.-J., Hahne J. M. & Müller K.-R. Real-time robustness evaluation of regression based myoelectric control against arm position change and donning/doffing. PLOS ONE 12, e0186318 (2017). 29095846 10.1371/journal.pone.0186318 PMC5667774

[26] Teh Y. & Hargrove L. J. Understanding Limb Position and External Load Effects on Real-Time Pattern Recognition Control in Amputees. IEEE Transactions on Neural Systems and Rehabilitation Engineering (2020) doi:10.1109/TNSRE.2020.2991643. PMC7391097 32396094

[27] Liu J., Zhang D., Sheng X. & Zhu X. Quantification and solutions of arm movements effect on sEMG pattern recognition. Biomedical Signal Processing and Control 13, 189–197 (2014).

[28] Hasbani M. H., Barsakcioglu D. Y., Jung M. K. & Farina D. Simultaneous and proportional control of wrist and hand degrees of freedom with kinematic prediction models from high-density EMG. Annu Int Conf IEEE Eng Med Biol Soc 2022, 764–767 (2022). 36085883 10.1109/EMBC48229.2022.9871346

[29] Simpetru R. C., Marz M. & Del Vecchio A. Proportional and Simultaneous Real-Time Control of the Full Human Hand From High-Density Electromyography. IEEE Trans Neural Syst Rehabil Eng 31, 3118–3131 (2023). 37440382 10.1109/TNSRE.2023.3295060

[30] Krasoulis A. & Nazarpour K. Discrete Action Control for Prosthetic Digits. IEEE Transactions on Neural Systems and Rehabilitation Engineering 30, 610–620 (2022). 35259109 10.1109/TNSRE.2022.3157710

[31] Pasquina P. F. First-in-man demonstration of a fully implanted myoelectric sensors system to control an advanced electromechanical prosthetic hand. Journal of Neuroscience Methods 244, 85–93 (2015). 25102286 10.1016/j.jneumeth.2014.07.016 PMC4317373

[32] Davis T. S. Restoring motor control and sensory feedback in people with upper extremity amputations using arrays of 96 microelectrodes implanted in the median and ulnar nerves. Journal of Neural Engineering (2016) doi:10.1088/1741-2560/13/3/036001. 27001946

[33] Salminger S. Long-term implant of intramuscular sensors and nerve transfers for wireless control of robotic arms in above-elbow amputees. Science Robotics (2019) doi:10.1126/scirobotics.aaw6306. 33137771

[34] Dewald H. A. Stable, three degree-of-freedom myoelectric prosthetic control via chronic bipolar intramuscular electrodes: A case study. Journal of NeuroEngineering and Rehabilitation (2019) doi:10.1186/s12984-019-0607-8. PMC6868792 31752886

[35] Ortiz-Catalan M., Hakansson B. & Branemark R. An osseointegrated human-machine gateway for long-term sensory feedback and motor control of artificial limbs. Science Translational Medicine (2014) doi:10.1126/scitranslmed.3008933. 25298322

[36] OrtizCatalan M., Mastinu E., Sassu P., Aszmann O. C. & Brånemark R. Self-contained neuromusculoskeletal arm prostheses. New England Journal of Medicine (2020) doi:10.1056/NEJMoa1917537. 32348644

[37] Lukyanenko P. Stable, simultaneous and proportional 4-DoF prosthetic hand control via synergy-inspired linear interpolation: a case series. Journal of NeuroEngineering and Rehabilitation 18, 50 (2021). 33736656 10.1186/s12984-021-00833-3 PMC7977328

[38] Tyler D. J. & Durand D. M. Chronic Response of the Rat Sciatic Nerve to the Flat Interface Nerve Electrode. Annals of Biomedical Engineering 31, 633–642 (2003). 12797612 10.1114/1.1569263

[39] Clark G. A., Ledbetter N. M., Warren D. J. & Harrison R. R. Recording sensory and motor information from peripheral nerves with Utah Slanted Electrode Arrays. in 2011 Annual International Conference of the IEEE Engineering in Medicine and Biology Society 4641–4644 (2011). doi:10.1109/IEMBS.2011.6091149. 22255372

[40] George J. A. Biomimetic sensory feedback through peripheral nerve stimulation improves dexterous use of a bionic hand. Science Robotics 4, eaax2352 (2019). 33137773 10.1126/scirobotics.aax2352

[41] Nguyen A. T. A bioelectric neural interface towards intuitive prosthetic control for amputees. Journal of Neural Engineering 17, 66001 (2020). 10.1088/1741-2552/abc3d3 33091891

[42] Cheng J., Yang Z., Overstreet C. K. & Keefer E. Fascicle-Specific Targeting of Longitudinal Intrafascicular Electrodes for Motor and Sensory Restoration in Upper-Limb Amputees. Hand Clin 37, 401–414 (2021). 34253313 10.1016/j.hcl.2021.04.004

[43] George J. A., Davis T. S., Brinton M. R. & Clark G. A. Intuitive neuromyoelectric control of a dexterous bionic arm using a modified Kalman filter. Journal of Neuroscience Methods (2020) doi:10.1016/j.jneumeth.2019.108462. 31711883

[44] Wendelken S. Restoration of motor control and proprioceptive and cutaneous sensation in humans with prior upper-limb amputation via multiple Utah Slanted Electrode Arrays (USEAs) implanted in residual peripheral arm nerves. Journal of NeuroEngineering and Rehabilitation (2017) doi:10.1186/s12984-017-0320-4. PMC5702130 29178940

[45] George J. A. Long-term performance of Utah slanted electrode arrays and intramuscular electromyographic leads implanted chronically in human arm nerves and muscles. Journal of Neural Engineering (2020) doi:10.1088/1741-2552/abc025. 33045689

[46] Dweiri Y. M. Stable Detection of Movement Intent From Peripheral Nerves: Chronic Study in Dogs. Proceedings of the IEEE 105, 50–65 (2017).

[47] Zbinden J. Improved control of a prosthetic limb by surgically creating electro-neuromuscular constructs with implanted electrodes. Science Translational Medicine 15, eabq3665. 37437016 10.1126/scitranslmed.abq3665

[48] Cheesborough J. E., Smith L. H., Kuiken T. A. & Dumanian G. A. Targeted muscle reinnervation and advanced prosthetic arms. Seminars in Plastic Surgery (2015) doi:10.1055/s-0035-1544166. PMC4317279 25685105

[49] Urbanchek M. G. Development of a Regenerative Peripheral Nerve Interface for Control of a Neuroprosthetic Limb. BioMed Research International 2016, 5726730 (2016). 27294122 10.1155/2016/5726730 PMC4886043

[50] Vu P. P. A regenerative peripheral nerve interface allows real-time control of an artificial hand in upper limb amputees. Science Translational Medicine (2020) doi:10.1126/scitranslmed.aay2857. PMC8082695 32132217

[51] Frost C. M. Regenerative peripheral nerve interfaces for real-time, proportional control of a Neuroprosthetic hand. Journal of NeuroEngineering and Rehabilitation (2018) doi:10.1186/s12984-018-0452-1. PMC6245539 30458876

[52] Hu Y. Regenerative peripheral nerve interface free muscle graft mass and function. Muscle & Nerve 63, 421–429 (2021). 33290586 10.1002/mus.27138

[53] Vu P. P. Long-term upper-extremity prosthetic control using regenerative peripheral nerve interfaces and implanted EMG electrodes. Journal of neural engineering 20, (2023). 10.1088/1741-2552/accb0c PMC10126717 37023743

[54] Kubiak C. A., Kemp S. W. P. & Cederna P. S. Regenerative peripheral nerve interface for management of postamputation neuroma. JAMA Surgery (2018) doi:10.1001/jamasurg.2018.0864. 29847613

[55] Lee C. Use of regenerative peripheral nerve interfaces and intramuscular electrodes to improve prosthetic grasp selection: A case study. Journal of neural engineering 19, (2022). 10.1088/1741-2552/ac9e1c PMC9942093 36317254

[56] Vaskov A. K. Surgically Implanted Electrodes Enable Real-Time Finger and Grasp Pattern Recognition for Prosthetic Hands. IEEE Transactions on Robotics 38, 2841–2857 (2022). 37193351 10.1109/tro.2022.3170720 PMC10168021

[57] Graham D., Bhardwaj P. & Sabapathy S. R. Secondary Thumb Reconstruction in a Mutilated Hand. Hand Clin 32, 533–547 (2016). 27712753 10.1016/j.hcl.2016.07.005

[58] Sandvall B. K., Keys K. A. & Friedrich J. B. Severe Hand Injuries From Fireworks: Injury Patterns, Outcomes, and Fireworks Types. J Hand Surg Am 42, 385.e1–385.e8 (2017). 10.1016/j.jhsa.2017.01.028 28341070

[59] Hooper R. C. Regenerative Peripheral Nerve Interfaces for the Management of Symptomatic Hand and Digital Neuromas. Plast Reconstr Surg Glob Open 8, e2792 (2020). 32766027 10.1097/GOX.0000000000002792 PMC7339232

[60] Senger J.-L. B. A Direct Comparison of Targeted Muscle Reinnervation and Regenerative Peripheral Nerve Interfaces to Prevent Neuroma Pain. Neurosurgery 93, 1180–1191 (2023). 37265342 10.1227/neu.0000000000002541

[61] Mauch J. T., Kao D. S., Friedly J. L. & Liu Y. Targeted muscle reinnervation and regenerative peripheral nerve interfaces for pain prophylaxis and treatment: A systematic review. PM&R 15, 1457–1465 (2023). 36965013 10.1002/pmrj.12972

[62] Dumanian G. A. Targeted Muscle Reinnervation Treats Neuroma and Phantom Pain in Major Limb Amputees: A Randomized Clinical Trial. Ann Surg 270, 238–246 (2019). 30371518 10.1097/SLA.0000000000003088

[63] Mioton L. M. Targeted Muscle Reinnervation Improves Residual Limb Pain, Phantom Limb Pain, and Limb Function: A Prospective Study of 33 Major Limb Amputees. Clin Orthop Relat Res 478, 2161–2167 (2020). 32452928 10.1097/CORR.0000000000001323 PMC7431223

[64] Culp C. J. & Abdi S. Current Understanding of Phantom Pain and its Treatment. Pain Physician 25, E941–E957 (2022). 36288580

[65] Subedi B. & Grossberg G. T. Phantom limb pain: mechanisms and treatment approaches. Pain Res Treat 2011, 864605 (2011). 22110933 10.1155/2011/864605 PMC3198614

[66] Ortiz-Catalan M. Phantom motor execution facilitated by machine learning and augmented reality as treatment for phantom limb pain: a single group, clinical trial in patients with chronic intractable phantom limb pain. Lancet (London, England) 388, 2885–2894 (2016). 27916234 10.1016/S0140-6736(16)31598-7

[67] Faghani Jadidi A., Stevenson A. J. T., Zarei A. A., Jensen W. & Lontis R. Effect of Modulated TENS on Corticospinal Excitability in Healthy Subjects. Neuroscience 485, 53–64 (2022). 35031397 10.1016/j.neuroscience.2022.01.004

[68] Page D. M. Motor Control and Sensory Feedback Enhance Prosthesis Embodiment and Reduce Phantom Pain After Long-Term Hand Amputation. Frontiers in Human Neuroscience 12, 1–16 (2018). 30319374 10.3389/fnhum.2018.00352 PMC6166773

[69] Ortiz-Catalan M. A highly integrated bionic hand with neural control and feedback for use in daily life. Science Robotics 8, eadf7360. 37820004 10.1126/scirobotics.adf7360

[70] Soghoyan G. Peripheral nerve stimulation enables somatosensory feedback while suppressing phantom limb pain in transradial amputees. Brain Stimulation: Basic, Translational, and Clinical Research in Neuromodulation 16, 756–758 (2023). 10.1016/j.brs.2023.04.017 37100202

[71] Gonzalez M. A. Electrical Stimulation of Regenerative Peripheral Nerve Interfaces (RPNIs) Induces Referred Sensations in People With Upper Limb Loss. IEEE Trans Neural Syst Rehabil Eng 32, 339–349 (2024). 38145529 10.1109/TNSRE.2023.3345164 PMC10938368

[72] Marasco P. D. Illusory movement perception improves motor control for prosthetic hands. Sci Transl Med 10, (2018). 10.1126/scitranslmed.aao6990 PMC5906050 29540617

[73] Vu P. Restoration of Proprioceptive and Cutaneous Sensation Using Regenerative Peripheral Nerve Interfaces in Humans with Upper Limb Amputations. Plastic & Reconstructive Surgery Publish Ah, (2022). 10.1097/PRS.0000000000009153 PMC9133017 35404335

[74] D’Anna E. A closed-loop hand prosthesis with simultaneous intraneural tactile and position feedback. Science Robotics (2019) doi:10.1126/scirobotics.aau8892. 33137741

[75] Raspopovic S., Valle G. & Petrini F. M. Sensory feedback for limb prostheses in amputees. Nature Materials 20, 925–939 (2021). 33859381 10.1038/s41563-021-00966-9

[76] Cuberovic I., Gill A., Resnik L. J., Tyler D. J. & Graczyk E. L. Learning of Artificial Sensation Through Long-Term Home Use of a Sensory-Enabled Prosthesis. Front Neurosci 13, 853 (2019). 31496931 10.3389/fnins.2019.00853 PMC6712074

